# Flat Bands Induced by Non‐Collinear Antiferromagnetism in CoBi_2_Te_4_

**DOI:** 10.1002/advs.76833

**Published:** 2026-07-31

**Authors:** Ziyuan Zhao, Yuefeng Yin, Jinxing Gu, Mark T. Edmonds, Nikhil V. Medhekar

**Affiliations:** ^1^ Department of Materials Science and Engineering Monash University Clayton Victoria Australia; ^2^ ARC Centre of Excellence in Future Low‐Energy Electronics Technologies FLEET Monash University Clayton Victoria Australia; ^3^ Department of Chemical and Biological Engineering Monash University Clayton Victoria Australia; ^4^ School of Physics and Astronomy Monash University Clayton Victoria Australia

**Keywords:** antiferromagnetism, atomic orbital, condensed matter physics, electronic band structure, fermi level, physics, spins, wannier function

## Abstract

The interplay of the topology of electronic wavefunctions with spin configurations in intrinsically magnetic topological materials causes various exotic electronic states, attracting much attention in condensed matter physics. Non‐collinear antiferromagnetic (*nc*AFM) state, characterized by spins lacking a specific orientation, remains enigmatic. Through first‐principles calculations and Wannier simulations, we predicted that the AB‐stacked CoBi_2_Te_4_ two‐septuple layer (2SL) hosts an intrinsically intralayer *nc*AFM state while preserving a band inversion between Bi‐*p* and Te‐*p* orbitals in its bulk band structure. Intriguingly, we identified a group of flat bands near the Fermi level in the edge state of (210) nanoribbon terminated by *nc*AFM coupling. These flat bands persist in the edge state of a CoBi_2_Te_4_‐Bi_2_Te_3_ heterostructure, where intralayer *nc*AFM coupling is maintained. In contrast, they vanish in systems lacking either band inversion or *nc*AFM coupling. This suggests that the formation of flat bands arises from the interplay between spin‐orbit coupling–induced band inversion and the spatially varying local exchange field generated by intralayer *nc*AFM ordering. Our findings not only uncover the topological properties and electronic states of an intrinsically *nc*AFM configuration for the first time but also provide a novel strategy for realizing flat bands, which could have implications for strongly correlated electron systems.

## Graphical abstract

Flat bands emerge at the edges of intrinsically non‐collinear antiferromagnetic CoBi_2_Te_4_ two‐septuple layers through the interplay of spin–orbit coupling–driven band inversion and non‐collinear antiferromagnetism, uncovering a previously unexplored mechanism for flat‐band formation and opening new opportunities for strongly correlated quantum states.

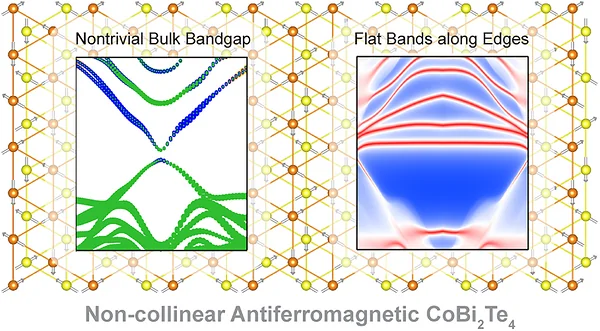

## 1 Introduction

Two‐dimensional (2D) topological insulators (TIs) are a unique class of electronic materials exhibiting conducting edge states that remain robust due to the protection of time reversal symmetry (TRS) [1, 2]. Interestingly, when magnetism is introduced, the TRS is broken, leading to the emergence of a variety of exotic topological phenomena, such as the quantum anomalous Hall effect (QAHE) [3, 4]. The introduction of magnetism in topological materials enables a novel platform to study the interplay between magnetism and topology, playing an important role in the discovery of novel electronic states.

Bi_2_Te_3_ is a well‐known topological insulator, where band inversion between Bi‐*p* and Te‐*p* orbitals, driven by a strong spin‐orbital coupling, serves as the key mechanism that swaps the conduction and valence bands at the Brillouin zone centre, enabling robust helical edge states protected by TRS [5]. Introducing magnetism, such as through doping with magnetic ions (e.g., Cr), breaks the TRS and can lead to exotic phenomena like the QAHE, though typically at ultralow temperatures [4, 6] (e.g., 30 mK in Cr‐doped (Bi_1−x_Sb_x_)_2_Te_3_ [4]). In contrast, *intrinsically* magnetic topological materials, such as MnBi_2_Te_4_, possess inherent magnetism with uniform magnetic distribution, enabling spin‐locked topological states at elevated temperatures [7, 8, 9, 10, 11, 12]. In MnBi_2_Te_4_, the half‐filled *d* orbital of Mn imparts a robust magnetism of 5 *μ*_B_ per formula unit, with a magnetic transition temperature of ∼25 K [11]. A zero‐field QAHE in a five–septuple‐layer (5SL) specimen of MnBi_2_Te_4_ can be observed at 1.4 K, and the quantization temperature can be further increased to 6.5 K with the application of an external magnetic field [9], marking a significant improvement over doped materials and positioning it as a promising platform for high‐temperature, low‐energy electronic devices.

Intrinsically magnetic topological materials can manifest a range of magnetic states, including ferromagnetic (FM), collinear antiferromagnetic (*c*AFM), and non‐collinear antiferromagnetic (*nc*AFM) states. The interplay between these magnetic states and topology can give rise to diverse and nontrivial electronic phenomena. For instance, in odd‐layered structures of MnBi_2_Te_4_, the presence of out‐of‐plane ferromagnetism induces the QAHE [10, 11], while the compensated interlayer antiferromagnetism in even layers disrupts edge states while preserving the integrity of an axion insulator [13]. Furthermore, theoretical calculations based on first principles have identified a class of compounds, denoted as XMnY (where X = Sr, Cu, Ba and Y = Sn, Pb), as TIs exhibiting intrinsically intralayer *c*AFM order with out‐of‐plane easy axis [14, 15]. The out‐of‐plane magnetization, with upward and downward orientations, engenders conducting states along the edge in both clockwise and counterclockwise directions, thereby resulting in a quantum spin Hall effect (QSHE).

On the other hand, the *nc*AFM state, characterized by non‐collinear magnetization vectors summing up to zero net magnetization, emerges as one of promising candidates for spintronic applications. Compared to FM materials, antiferromagnets possess inherent advantages, such as negligible stray fields and robustness against external magnetic perturbations. Moreover, the non‐collinear spin ordering inherent in *nc*AFM states introduces scattering mechanisms distinct from those observed in collinear magnets, holding the potential for unveiling extraordinary magnetoelectric phenomena, including the induction of anomalous Hall effects, as demonstrated in recent studies [16, 17]. In addition, the non‐collinear arrangement of magnetic moments generates a spatially varying local exchange field at the atomic scale, providing an additional degree of freedom for tailoring the electronic structure and realizing novel quantum phenomena.

Flat bands, where electron energies are confined within a narrow energy window [18], have become a focal point in condensed matter physics due to their association with strong electron correlations [19, 20] and the emergence of associated exotic quantum states. Achieving flat bands often requires specific structural and electronic arrangements that suppress dispersion, such as in Kagome lattice [21, 22, 23, 24] and Moiré superlattices [25, 26, 27, 28, 29]. In Kagome lattices, the geometric frustration of the lattice induces destructive interference, flattening the band structure. Similarly, in Moiré superlattices, the lattice mismatch and small twist angles create periodic potentials that confine electron motion and yield flat bands at specific twist angles. Another pathway to realize flat bands is through the introduction of magnetic domain walls. Recent calculations predicted topological one‐dimensional flat bands in *c*AFM TIs based on V compounds (i.e., VBi_2_Te_2_Se_2_, VBi_2_Te_4_, and VSb_2_Te_4_) [30], which share the same crystal structure as MnBi_2_Te_4._ In that work, the authors considered magnetic domain walls on the (0001) surface and demonstrated that the resulting real‐space variation of the local magnetization can induce localized topological flat bands. These findings suggest that magnetic order provides an additional route for engineering flat bands.

So far, the intralayer *nc*AFM state has been identified in metallic Mn Kagome lattices, exhibiting a large anomalous Hall conductivity [31, 32, 33]. However, attempts to introduce a bandgap in these Mn Kagome lattices have proven unsuccessful. The realization of intrinsically magnetic topological insulators (TIs) featuring the *nc*AFM state remains elusive, and the exploration of associated topological properties and electronic states is still in its early stages. The discovery of MnBi_2_Te_4_ has opened a promising avenue for investigating other divalent transition metals. For instance, a series of *c*AFM TIs with in‐plane magnetization have been predicted in V‐based compounds [30]. And FM TIs can be achieved in Cr‐based compounds induced by Jahn‐Teller Effect [34]. Notably, our recent studies combining first principles calculations and Monte Carlo simulations have revealed that a septuple layer of CoBi_2_Te_4_ stabilizes in an intrinsically *nc*AFM state with a Néel temperature of 10 K [35]. The discovery of an unconventional non‐collinear configuration provides a foundation for further exploration of novel magnetoelectric phenomena.

Herein, we systematically investigated the magnetic, electronic, and topological properties of CoBi_2_Te_4_ films using first‐principles calculations and Wannier interpolation. Both one‐septuple layer (1SL) and two‐septuple layer (2SL) structures of CoBi_2_Te_4_ exhibit ground magnetic state of *nc*AFM states with an in‐plane easy axis. The CoBi_2_Te_4_ 2SL in AB‐stacked configuration features a nontrivial bulk bandgap, characterized by a band inversion between Bi‐*p* and Te‐*p* orbitals, and notably hosts a set of flat bands near the Fermi level in its edge states. To elucidate the origin of these flat bands, we examined two alternative cases: AA‐stacked CoBi_2_Te_4_ 2SL with an *nc*AFM state but without band inversion, and AB‐stacked CoBi_2_Te_4_ 2SL with a collinear magnetic state while preserving band inversion. In both scenarios, we found that the flat bands disappear. This indicates that the emergence of flat bands relies on the interplay between nontrivial band inversion and intralayer *nc*AFM coupling. Our findings provide new insights into the formation of flat bands in intrinsically magnetic topological materials.

## 2 Results and Discussion

We initiated our investigation by examining the thermodynamic stability and electronic band structure of CoBi_2_Te_4_ 1SL characterized by an intralayer *nc*AFM state (Figure 1a). Molecular dynamics (MD) simulations were performed using a $\sqrt{3}$ × $\sqrt{3}$ supercell of *nc*AFM CoBi_2_Te_4_ 1SL at the temperature of 300 K. As illustrated in Figure S1, after 10 ps the free energy quickly equilibrates and fluctuates around a stable value. The final snapshot indicates that the structure retains its overall integrity without noticeable bond breakage, indicating its thermodynamic stability. Band structure analysis reveals that, in the absence of spin‐orbit coupling (SOC), the conduction band minimum (CBM) arises from contributions by both Co‐*d* and Bi‐*p* orbitals, while the valence band maximum (VBM) is primarily influenced by Te‐*p* bands, as illustrated in Figure 1b. Upon considering SOC, Co‐*d* bands persist in the high‐energy region, whereas the Bi‐*p* states within the conduction bands shift downward toward the Fermi level, resulting in a reduced bandgap (Figure 1c). The pronounced change upon including SOC arises from the strong SOC effect on the Bi‐*p* and Te‐*p* bands. Specifically, at the PBE level of theory, the bandgap decreases from 1.05 eV (without SOC) to 0.32 eV (with SOC), identifying CoBi_2_Te_4_ 1SL as an indirect insulator. We also performed HSE calculations, which yield larger bandgaps of 1.37 and 0.59 eV with and without SOC, respectively, while preserving the overall band structure shape (Figure S2). Furthermore, the calculation of topological invariants reveals a Chern number of zero, indicating its topological triviality.

**FIGURE 1:**
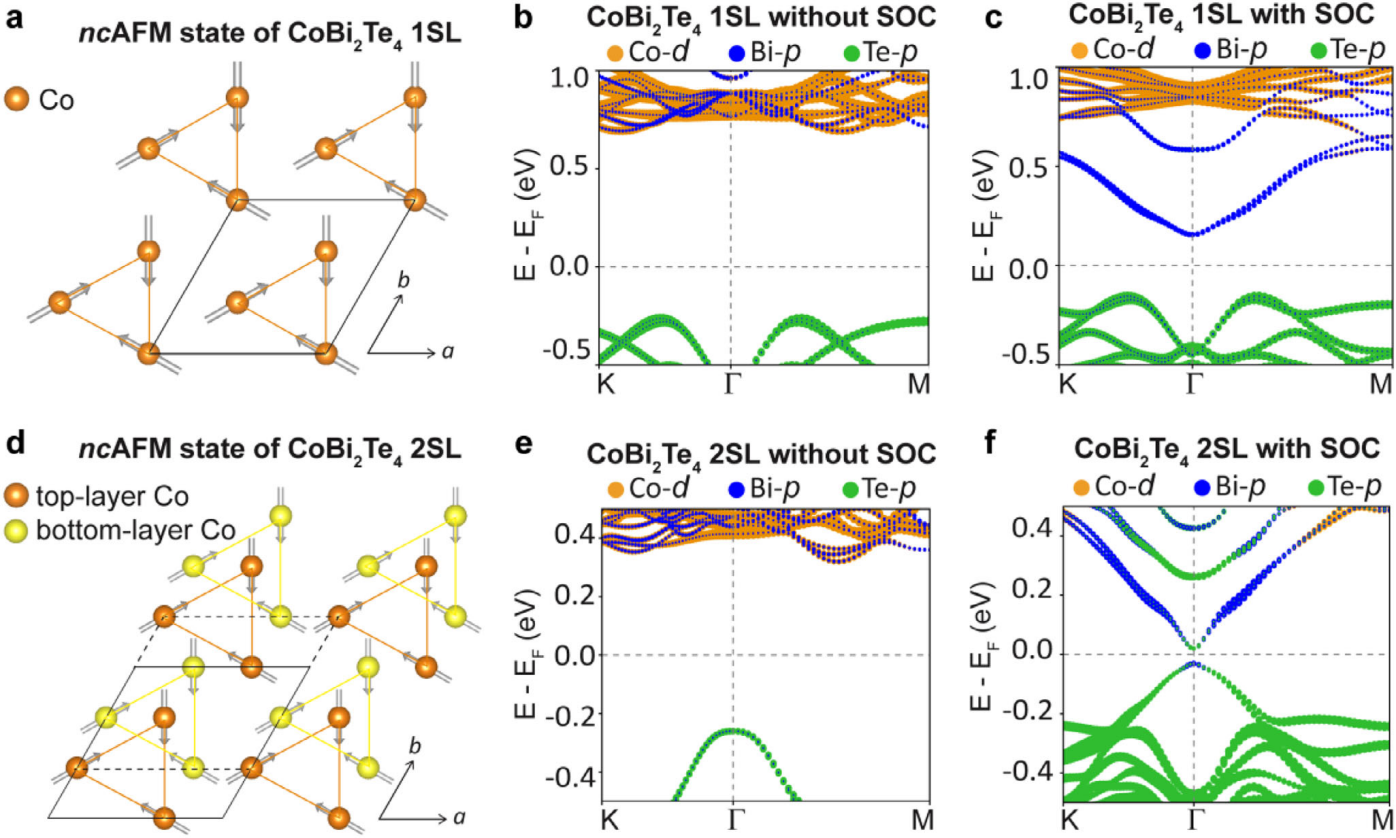
Ground magnetic states and band structures of CoBi_2_Te_4_ 1SL and 2SL. (a) The top view of the intralayer *nc*AFM configuration of CoBi_2_Te_4_ 1SL with $\sqrt{3}$ × $\sqrt{3}$ *R*30° in‐plane supercell containing three Co atoms. Band structures with orbital characters of CoBi_2_Te_4_ 1SL at the PBE level of theory excluding (b) and including SOC (c). (d) The top view of the intralayer *nc*AFM configuration in AB‐stacked CoBi_2_Te_4_ 2SL. The orange atoms are the Co of top layer, and the yellow atoms are the Co of bottom layer. The bottom layer is stacked in the (0, $\frac{1}{3}$) direction of the top layer. Band structures with orbital characters of CoBi_2_Te_4_ 2SL at the PBE level of theory excluding (e) and including SOC (f).

The band topology of MnBi_2_Te_4_‐family materials strongly depends on layer stacking, since interlayer coupling modifies the electronic structure, particularly near the Fermi level [10, 11, 36, 37]. We investigated the stacking configurations of CoBi_2_Te_4_ 2SL, considering two arrangements: AA and AB stacking (Figure S3). The AA‐stacked configuration is formed by aligning the atoms in the top layer directly above the corresponding atoms in the bottom layer. In contrast, the AB‐stacked CoBi_2_Te_4_ 2SL was constructed by shifting the bottom layer along the (0, $\frac{1}{3}$) direction relative to the top layer. After full structural relaxation, the AA‐stacked configuration exhibits an interlayer distance of 3.73 Å, while the AB‐stacked configuration stabilizes at a smaller interlayer distance of 2.70 Å. By comparing their total energies, we discerned that AB stacking is energetically more favourable, with a total energy 13.29 meV/atom lower than AA stacking. Detailed structural parameters of both AA‐ and AB‐stacked configurations of CoBi_2_Te_4_ 2SL are provided in Table S1. Given its greater stability, we selected the AB‐stacked configuration as the focus of this work.

Analysis of the *d*‐orbitals of Co atom in AB‐stacked CoBi_2_Te_4_ 2SL from the projected density of states (PDOS) reveals a high‐spin state (see Figure S4). Five of the seven *d*‐electrons occupy spin‐up states (one per orbital), while the remaining two occupy spin‐down levels, mainly filling the lower‐energy t_2g_ orbitals, which is in line with octahedral ligand field splitting. As a result, Co exhibits a magnetic moment of 3 *μ*_B_, which remains essentially unchanged compared to the 1SL structure [35]. To ascertain the global magnetic ground state of the AB‐stacked CoBi_2_Te_4_ 2SL, we explored three collinear magnetic configurations, including ferromagnetic (FM), interlayer collinear antiferromagnetic (inter_*c*AFM), and intralayer collinear antiferromagnetic (intra_*c*AFM) states, and two non‐collinear magnetic states, i.e., intralayer non‐collinear antiferromagnetic (*nc*AFM) and non‐coplanar antiferromagnetic (*ncp*AFM) states. The *nc*AFM configuration is illustrated in Figure 1d, and schematic representations of the others can be found in Figure S5. All considered collinear and non‐collinear configurations adopt in‐plane spin orientations, consistent with the magnetic anisotropy energy (MAE) results discussed below. The relative energies of these magnetic states are summarized in Table S2, which clearly indicates that the CoBi_2_Te_4_ 2SL favors a *nc*AFM ground state.

We employed the Heisenberg model for the Co lattice to calculate the exchange coupling parameter between nearest neighbouring Co atoms (*J*), revealing a large value of −1.46 meV. Additionally, to examinate the MAE, we conducted spin‐orbital coupling (SOC) calculations on CoBi_2_Te_4_ 2SL, evaluating the magnetization along three directions, i.e., [100], [010], and [001]. The energy minima along [100] and [010] suggest an in‐plane easy axis, and the estimated MAE is −0.05 meV per Co atom. Based on the exchange coupling parameter and the MAE, we performed Monte Carlo simulations to probe the Néel temperature of CoBi_2_Te_4_ 2SL. In comparison with CoBi_2_Te_4_ 1SL with a Néel temperature of ∼10 K [35], CoBi_2_Te_4_ 2SL exhibits an enhanced Néel temperature of ∼ 20 K (Figure S6). This increase aligns well with the enhanced exchange coupling parameter in CoBi_2_Te_4_ 2SL (*J* = −1.46 meV), compared to the weaker coupling in the 1SL case (*J* = −0.82 meV).

Analysing the electronic band structure of AB‐stacked CoBi_2_Te_4_ 2SL without considering SOC reveals an insulating electronic state with an indirect bandgap of 0.57 eV (Figure 1e). Similar to the CoBi_2_Te_4_ 1SL, the conduction bands in the CoBi_2_Te_4_ 2SL originate from both Co‐*d* and Bi‐*p* orbitals in the absence of SOC, and the valence bands are primarily composed of Te‐*p* orbitals. Upon considering SOC, it results in a notable shift: the Bi‐*p* bands move downward, and Te‐*p* bands move upward, leading to a significant reduction in the bandgap, ultimately forming a 52 meV direct bandgap at Γ point (Figure 1f). Meanwhile, the reduction in the bandgap is accompanied by a discernible band inversion. The Bi‐*p* band undergoes inversion toward the Γ point of the VBM, while the Te‐*p* bands becomes dominant at the Γ point of the CBM. We also examined the band structures for *U* = 2 and 6 eV to assess the Hubbard *U* dependence of the Co *d*‐bands. As shown in Figure S7, the Co‐*d* conduction bands shift downward at *U* = 2 eV and upward at *U* = 6 eV. In contrast, the Bi‐*p* and Te‐*p* bands remain largely unaffected by varying *U* values and continue to define the CBM and VBM, preserving the band inversion in the 2SL structure. Band inversion is usually associated with nontrivial topological phases, and 2D magnetic topological insulators hold intriguing possibility for achievement of Chern insulators. To investigate this possibility, we calculated the Wannier charge center (WCC) on the closed momentum plane with the k*_z_* = 0 for the AB‐stacked CoBi_2_Te_4_ 2SL. As illustrated in Figure S8, the resulting zero Chern number (*C* = 0) indicates that CoBi_2_Te_4_ 2SL does not exhibit Chern insulator behavior, despite the presence of band inversion and broken TRS. This highlights that band inversion and TRS breaking are necessary but not sufficient for realizing a Chern insulator phase. An additional key requirement is net magnetization, which provides a uniform magnetic field capable of supporting chiral edge states. Unlike FM MnBi_2_Te_4_, where all magnetic moments are aligned to produce a net magnetization, the *nc*AFM CoBi_2_Te_4_ phase features a non‐collinear spin texture with zero net magnetization. Although TRS is broken locally in the *nc*AFM state, the global cancellation of spin orientations prevents the formation of a uniform magnetic field, precluding the formation of chiral edge modes and Chern insulator behaviors. Nontrivial bulk band inversion in 2D systems can give rise to novel edge states, offering a rich platform for exploring unconventional electronic phenomena. The identification of an intrinsically intralayer *nc*AFM configuration in CoBi_2_Te_4_ 2SL further motivates an in‐depth investigation of the corresponding edge states. Using *nc*AFM CoBi_2_Te_4_ 2SL as a model system, we examined how different non‐collinear magnetic orderings at edge terminations influence the edge band structure. The remaining part of this work focuses on the unique edge states arising from the *nc*AFM configurations.

We first explored the edge states of CoBi_2_Te_4_ 2SL nanoribbons terminated along the (100) and (010) crystallographic directions, as shown in Figure 2a–d. In these two nanoribbon structures, despite sharing a similar atomic configuration, each termination exhibits a distinct magnetic order. Specifically, the (100)‐edge termination (Figure 2a,b) displays a ferromagnetic state, with the outermost exposed Co atoms aligning in the same magnetization direction, forming what we term as the *c*FM edge. Conversely, as shown in Figure 2c,d, the (010) nanoribbon exhibits outermost exposed Co atoms with two distinct magnetization directions at the edge termination, resulting in a non‐zero net magnetic moment and forming *nc*FM edges. For both *c*FM and *nc*FM nanoribbons, we conducted electronic state calculations on both left and right sides. Intriguingly, in each edge state, we observed a gapped Dirac point at the Γ point, with a small bandgap of approximately 0.01 eV (Figure 2e–h). These gapped electronic edge states underscore the non‐metallic nature of the (100) and (010) nanoribbons and further corroborate our earlier discussion regarding the absence of Chern insulator behaviour in CoBi_2_Te_4_ 2SL.

**FIGURE 2:**
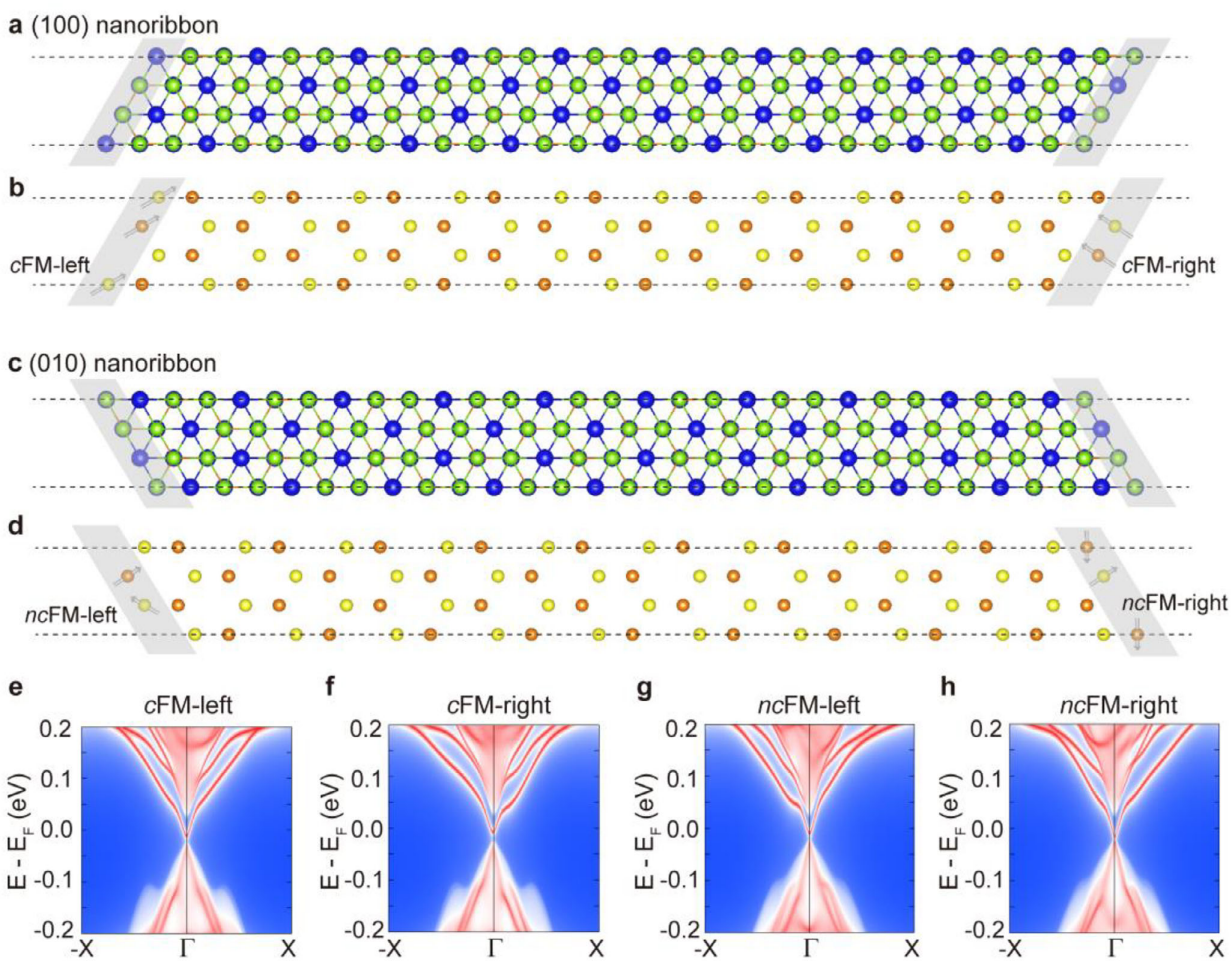
The top views, magnetic configurations, and edge band structures of (100) and (010) nanoribbons in CoBi_2_Te_4_ 2SL. (a, c) The top views of the atomic structures of the (100)‐edge and (010)‐edge nanoribbons, respectively. (b, d) The top views of the arrangement of Co atoms in the (100)‐edge and (010) nanoribbons, respectively. Orange and yellow colors denote Co atoms from the top and bottom SL, respectively, the arrows represent the magnetization directions of Co atoms. (e‐h) The edge band structures of the specific edges.

Additionally, we explored the possibility of another high‐symmetry nanoribbon orientation along the (210) direction, as illustrated in Figure 3a. To assess its stability, we conducted DFT calculations to relax the edge structure of the (210) nanoribbon. The result, presented in Figure S9, revealed that the edge atoms retained their bulk shape without any apparent reconstruction after relaxation. To evaluate the thermodynamic stability of different edge terminations, we calculated the specific edge energies [38, 39] of the (100), (010), and (210) nanoribbons (see Table S3). The (210) edge yields a lower specific edge energy (0.65 eV/Å), compared to 0.72 and 0.75 eV/ Å for the (010) and (100) edges, respectively. This result suggests that the (210) termination is energetically competitive and may be locally favorable under certain conditions. We note that stabilization of high‐index surfaces has also been reported in various systems [40, 41, 42]. From an experimental perspective, although high‐index terminations are unlikely to be obtained via simple mechanical cleavage, they may be realized through controlled synthesis strategies. For example, epitaxial growth on vicinal substrates can template kinked geometries associated with high‐index orientations [43, 44, 45]. Alternatively, bottom‐up growth under controlled chemical potentials may stabilize kinked edges during the growth process [46, 47].

**FIGURE 3:**
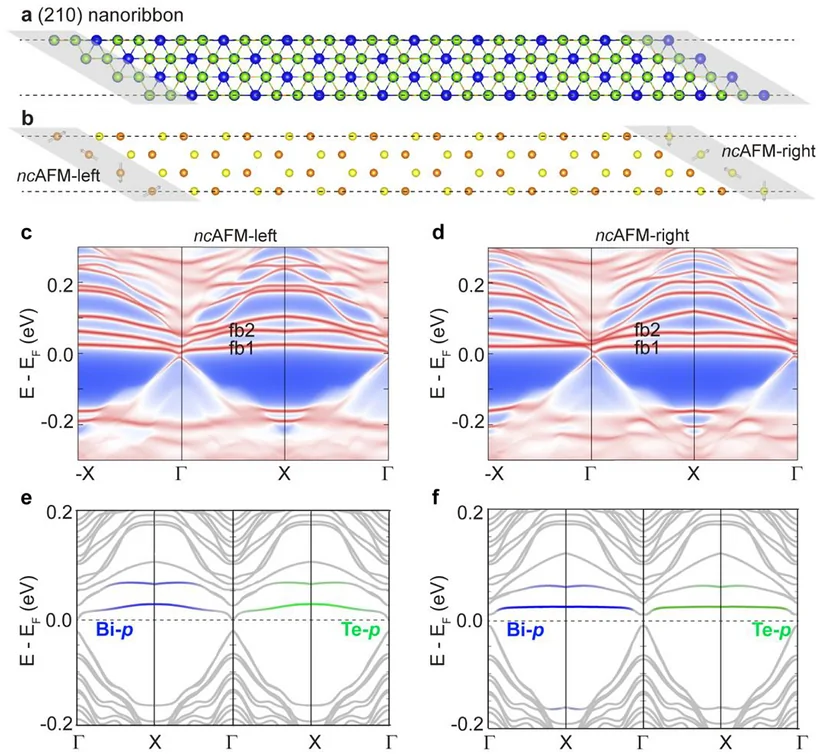
The top views, magnetic configurations, and edge band structures of CoBi_2_Te_4_ 2SL (210) nanoribbon. (a) The top view of the atomic structure of the (210) nanoribbon. (b) The top view of the arrangement of Co atoms arrangement in the (210) nanoribbon. The edge states of the left and right sides for *nc*AFM edge are shown in (c) and (d), respectively. The flat bands in the edge states are labelled as fb1 and fb2. The edge band structures with Bi‐*p* (blue) and Te‐*p* (green) orbital characterization of the left and right sides for *nc*AFM edge are shown in (e) and (f), respectively. In (b), orange and yellow colors denote Co atoms from the top and bottom SL, respectively; the arrows represent the magnetization directions of Co atoms.

In the (210)‐edge terminations, we observed three different magnetization directions for the outermost exposed Co atoms on both the left and right sides, as depicted in Figure 3b. The total magnetic moments of the exposed Co atoms on each side sum to zero, indicating a non‐collinear antiferromagnetic coupling, which we term the *nc*AFM edge. We employed Wannier interpolation to derive the edge states at the *nc*AFM terminations. Notably, two flat bands, labeled fb1 and fb2, emerge near the Fermi level within the bulk bandgap, as depicted in Figure 3c,d. The fb1 band is characterized by its straight trajectory traversing the entire XΓ/‐XΓ path, with only a slight twist near the Γ point. It closely interacts with the valence band maximum (VBM), forming a gapped Dirac point near the Γ point. fb2, located above fb1, shows slight bending at the Γ point while maintaining an overall flat‐band characteristic. To quantitatively evaluate their flatness, we calculated the bandwidths of these flat bands (Table S4). The fb1 and fb2 bands show narrow bandwidths of 23.91 meV and 38.53 meV at the left edge, and 17.63 meV and 37.46 meV at the right edge, respectively. These values are significantly smaller than the bulk bandgap (∼52 meV), indicating a strong suppression of kinetic energy and a pronounced flat‐band character. To further characterize the quality of these flat bands, we also evaluated the flatness ratio. As summarized in Table S4, all flat bands exhibit flatness ratios larger than 1, with the fb1 band at the right edge reaching a value of 2.95. This value exceeds the flatness ratios reported for many representative Kagome flat‐band systems (typically ∼1) [23, 24, 48], indicating that the flat bands in CoBi_2_Te_4_ are not only weakly dispersive but also well isolated from the neighboring dispersive edge bands. Despite the distinct exposure of the outermost Co atoms on the left and right sides of the nanoribbon, corresponding to the upper and lower layers of the material, both edges display characteristic features of *nc*AFM coupling. As a result, the electronic states on both sides of the nanoribbon show remarkable similarities. To further verify the robustness of these flat bands, we performed additional calculations with Hubbard *U* values of 2 and 6 eV. The results shown in Figure S10 confirm that the essential features of the flat bands are well preserved.

Since the flat band appears exclusively in the (210) edge spectrum and is absent in the bulk band structure, it can be naturally attributed to edge‐derived electronic states. To further clarify the physical origin and localization characteristics of the flat‐band edge states, we take the right edge of the (210) nanoribbon as an example and investigate the local density of states (LDOS) evolution from the outermost edge region toward the inner layers. Here, we group every three atomic layers into a single slab unit. The slabs are numbered sequentially from the edge toward the bulk as s_1, s_2, s_3, etc. As shown in Figure 4, the calculated LDOS map exhibits pronounced resonance peaks at the flat‐band energies near the outermost edge region. These resonance features gradually weaken as the probing position moves away from the edge and eventually merge into the bulk background, indicating the strongly localized nature of the flat‐band edge states. The slab‐resolved spectral evolution therefore establishes a clear correspondence between the flat‐band states and their edge origin. The calculated LDOS distribution may also provide useful guidance for future experimental characterization, such as scanning tunneling microscopy/spectroscopy measurements.

**FIGURE 4:**
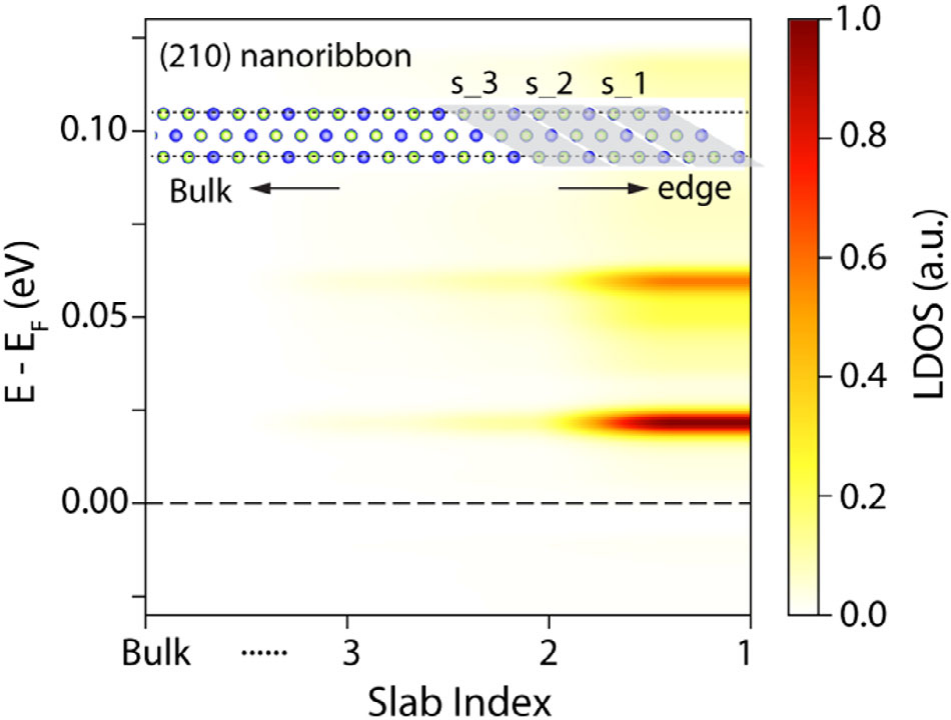
Spatially resolved local density of states (LDOS) of the (210) nanoribbon right edge. The LDOS is projected onto successive atomic regions extending from the edge toward the bulk. Pronounced resonance peaks associated with flat‐band states appear near E – E_F_ ≈ 0.02 and 0.06 eV. The spectral intensity is maximized at the outermost edge region (s_1) and decays rapidly toward the interior regions (s_2 and s_3), indicating strong spatial localization of the edge states. Inset: Atomic structure of the (210) nanoribbon and the partition scheme used for the LDOS projection, with regions labelled s_1, s_2, s_3, where s_1 corresponds to the edge and increasing indices indicate progression toward the bulk.

Upon analyzing the orbital characteristics of the flat bands at the *nc*AFM edges, we found that these flat bands are primarily composed of the Bi‐*p* and Te‐*p* orbitals, as illustrated in Figure 3e,f, which aligns with the band inversion between Bi‐*p* and Te‐*p* orbitals. To further clarify the role of band inversion in flat‐band formation, we adopted AA‐stacked CoBi_2_Te_4_ 2SL as a representative topologically trivial case for comparison with the nontrivial AB‐stacked structure. As shown in Figure S11b, the AA‐stacked structure exhibits a trivial bulk bandgap of 0.20 eV without band inversion. In this stacking configuration, the relatively weak interlayer coupling suppresses the orbital hybridization responsible for the band inversion, thereby maintaining a topologically trivial electronic structure [36, 37]. For the corresponding (210) nanoribbon (Figure S12), no edge bands appear within the bulk bandgap, in sharp contrast to the AB‐stacked case. Although the AA‐stacked CoBi_2_Te_4_ 2SL still possesses *nc*AFM ordering, the absence of band inversion prevents the emergence of flat bands. These results demonstrate that band inversion is responsible for the emergence of flat bands along edges.

On the other hand, the dispersion of flat bands is strongly influenced by the unique features of the *nc*AFM terminations. To examine the role of *nc*AFM coupling, we constructed an artificial (210) nanoribbon of the AB‐stacked CoBi_2_Te_4_ 2SL with collinear magnetic states, as shown in Figure S13a. In this model, Co atoms at the left edge exhibit magnetic moment directions forming 30° angles with the edge, while those on the right form 90° angles. Along the edges with collinear magnetism, additional bands appear with in the bandgap but are shifted away from the Fermi level (Figure S13b,c). Specifically, the flat bands near the Fermi level, preserved in the *nc*AFM edges, undergo significant bending in the presence of collinear magnetism. These results indicate that *nc*AFM ordering plays a crucial role in stabilizing the nearly dispersionless flat bands. Taken together, the flat bands arise from cooperative contributions of different orbitals. The Bi‐*p* and Te‐*p* states directly form the essential basis of the flat bands, consistent with the band inversion in the bulk band structure. Meanwhile, Co‐*d* orbitals, through the non‐collinear magnetic configuration of the Co layers, play a crucial role in shaping the nearly dispersionless character and thus contribute indirectly to the flat bands. This cooperative interplay between Bi/Te‐*p* and Co‐*d* states underpins the formation and robustness of the flat bands.

The *nc*AFM state in CoBi_2_Te_4_ 2SL is complicated, featuring both intralayer and interlayer magnetic couplings (Figure 1d). The resulting non‐collinear spin configuration is therefore determined by the combined effect of these two magnetic interactions. To clarify their respective roles in the formation of the flat bands, we constructed a CoBi_2_Te_4_‐Bi_2_Te_3_ heterostructure by juxtaposing a CoBi_2_Te_4_ 1SL and a Bi_2_Te_3_ one quintuple layer (1QL) (refer to Figure 5a). The CoBi_2_Te_4_‐Bi_2_Te_3_ heterostructure maintains good structural compatibility between layers due to a minimal lattice mismatch of ∼1%. By comparing the total energies of various magnetic states, including FM, intra_*c*AFM, *nc*AFM, and *ncp*AFM, it is evident that the CoBi_2_Te_4_‐Bi_2_Te_3_ heterostructure consistently favours the *nc*AFM state as the ground magnetic state (see Table S2 in Supporting Information). Since the CoBi_2_Te_4_‐Bi_2_Te_3_ heterostructure contains only a single Co layer, the ground magnetic state *nc*AFM is primarily governed by intralayer magnetic couplings between Co atoms, while interlayer magnetic interactions remain negligible.

**FIGURE 5:**
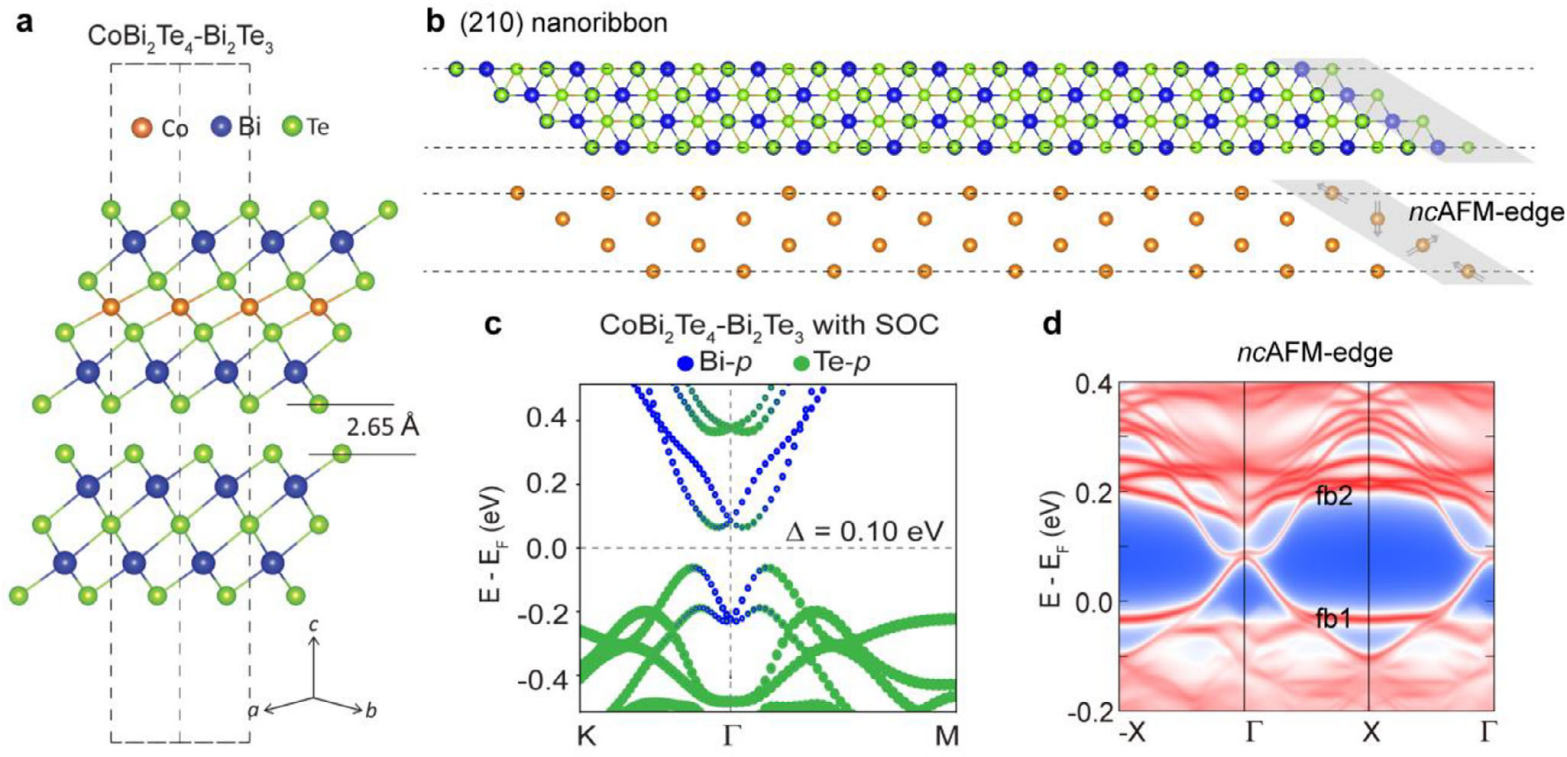
The side view, band structure, and edge state of CoBi_2_Te_4_‐Bi_2_Te_3_ heterostructure. (a) The side view of the CoBi_2_Te_4_‐Bi_2_Te_3_ heterostructure, which stabilizes in a AB‐stacking configuration with a interlayer distance of 2.65 Å. (b) The top views of the atomic structure and magnetic configuration of the *nc*AFM‐edge nanoribbon. (c) Band structure with Bi‐*p* and Te‐*p* orbital characterization of CoBi_2_Te_4_‐Bi_2_Te_3_ heterostructure at the PBE level of theory including SOC. (d) The edge band structure of the *nc*AFM edge of the CoBi_2_Te_4_‐Bi_2_Te_3_ heterostructure. The flat bands in the edge states are labelled as fb1 and fb2.

Examining the band structure of the CoBi_2_Te_4_‐Bi_2_Te_3_ heterostructure, depicted in Figure S14 and Figure 5c, reveals an insulating electronic state characterized by an indirect bandgap of 0.66 and 0.10 eV at the PBE level without and with consideration of SOC, respectively. Similar to the case in AB‐stacked CoBi_2_Te_4_ 2SL, the inclusion of SOC induces a band inversion between Bi‐*p* and Te‐*p* orbitals in the heterostructure. Unlike the 2SL system, the heterostructure exhibits additional band splitting around Γ point, where Rashba effect emerges due to structural inversion asymmetry at the interface [49, 50]. Upon cutting a nanoribbon along the (210) direction, the exposed Co atoms at the termination exhibit distinct magnetization directions, maintaining an intralayer *nc*AFM coupling (as depicted in Figure 5b). Flat bands, identified as fb1 and fb2 in Figure 5d, persist in the edge state, though they exhibit greater bending at the Γ point. Specifically, fb1 resides below the Fermi level, displaying flat characteristics around the X/‐X point while exhibiting bending near the Γ point. Similarly, fb2 lies above the Fermi level, showcasing flat attributes around the X/‐X point but displaying disruptions around the Γ point. These findings underscore the significance of intralayer *nc*AFM coupling in facilitating the appearance of flat bands in the CoBi_2_Te_4_‐Bi_2_Te_3_ heterostructure.

Recently, a *nc*AFM ground state has been predicted in the FeBi_2_Te_4_ system, which exhibits a similar in‐plane non‐collinear magnetic ordering [51]. The *nc*AFM FeBi_2_Te_4_ 2SL is found to have a bandgap of approximately 10 meV. Our band structure calculations reveal that it hosts SOC‐induced band inversion between Bi‐*p* and Te‐*p* orbitals, as shown in Figure S15a. Given that it possesses the key ingredients for flat‐band formation, i.e., the *nc*AFM state and band inversion, we further investigated its edge states. Notably, flat bands emerge near the Fermi level along the (210) edge (Figure S15b). The appearance of analogous flat bands in the FeBi_2_Te_4_ system suggests that this phenomenon is not unique to CoBi_2_Te_4_, but it may represent a general feature of *nc*AFM Bi_2_Te_3_‐based materials.

The emergence of flat bands in *nc*AFM CoBi_2_Te_4_ systems arises from the interplay between SOC‐induced band inversion, non‐collinear antiferromagnetism, and reduced dimensionality. In Bi_2_Te_3_‐based materials, SOC typically drives band inversion between Bi‐*p* and Te‐*p* orbitals, resulting in distinguished electronic states such as topological edge modes. In the case of *nc*AFM CoBi_2_Te_4_, the presence of non‐collinear magnetic order generates a spatially varying local exchange field, which suppresses the dispersion of the Bi/Te‐*p*‐derived states through exchange coupling between the Co‐*d* and Bi/Te‐*p* orbitals, thereby stabilizing the nearly dispersionless flat bands. Reduced dimensionality further promotes the localization of these topological edge states in the (210) nanoribbon, facilitating the observation of the flat‐band character. This mechanism fundamentally differs from previously reported flat‐band systems. In Kagome lattices, flat bands originate from geometric frustration that suppresses the effective electron hopping [21, 23], whereas in moiré superlattices they arise from long‐wavelength periodic potentials induced by lattice mismatch and small twist angles [25, 26]. In contrast, the flat bands in CoBi_2_Te_4_ emerge through the cooperative interplay between SOC‐induced band inversion and the *nc*AFM ordering, representing an unconventional route to flat‐band formation driven by the combined effects of topology and magnetism.

## 3 Conclusions

In summary, AB‐stacked CoBi_2_Te_4_ 2SL was predicted to host intrinsically *nc*AFM state with a Néel temperature of approximately 20 K and an insulating bulk electronic states characterized by nontrivial band inversion. Remarkably, we demonstrated the appearance of flat bands in the edge states of CoBi_2_Te_4_ films. Specifically, in the CoBi_2_Te_4_ 2SL nanoribbon, these flat bands display a straight trajectory spanning the entire XΓ/‐XΓ path and reside near the Fermi level. Similar flat bands persist in the edge state of a CoBi_2_Te_4_‐Bi_2_Te_3_ heterostructure, albert with a bending around Γ point. These edge‐localized flat bands exhibit a high density of states and suppressed dispersion near the Fermi level, creating an ideal platform to explore strong correlation effects, such as Luttinger liquids and charge density waves [52, 53, 54]. In addition, the nearly dispersionless flat‐band edge states, characterized by a strongly reduced group velocity, may significantly suppress carrier transport along the (210) direction relative to other crystallographic directions. This direction‐dependent suppression of transport could give rise to pronounced anisotropy in the transport behavior of CoBi_2_Te_4_ [55, 56]. Our work not only advances the understanding of *nc*AFM‐driven electronic phenomena but also expands the materials phase space for discovering emergent correlated states.

### 3.1 Computational Methods

Our calculations of structural optimization, electronic and magnetic properties were performed using density functional theory (DFT) [57], employing the generalized gradient approximation (GGA) [58] as implemented in the VASP code [59, 60]. To prevent interactions between adjacent layers, a vacuum spacing of approximately 15 Å was introduced. Additionally, van der Waals interactions were accounted for using the DFT‐D3 method [61, 62]. To correct the on‐site electron‐electron interaction of the transition metal, we incorporated the Hubbard *U* correction [63], with a value of *U* = 4 eV applied to the Co 3*d*‐states. A plane‐wave basis set with a cutoff energy of 500 eV was utilized for all calculations. To ensure a good convergence, the Monkhorst‐Pack *k* mesh of 12 × 12 × 1 and 7 × 7 × 1 were used for one unit cell and $\sqrt{3}$ × $\sqrt{3}$ in‐plane supercell, respectively. Magnetocrystalline anisotropy energies (MAE) were calculated by using dense *k*‐point mesh of 21 × 21 × 1. Chern number and edge states were calculated based on maximally localized Wannier functions as implemented in WannierTools package [64, 65]. First‐principles molecular dynamics (MD) simulations in NVT ensemble were performed using the Andersen heat bath method lasted for 9 ps with a time step of 0.5 fs [66]. Further details on the computational procedures are provided in the Supporting Information.

## Author Contributions

**Mark T. Edmonds**: supervision, conceptualization. **Ziyuan Zhao**: conceptualization, investigation, writing – original draft, methodology, validation, visualization, writing – review and editing, formal analysis, data curation. **Jinxing Gu**: methodology, visualization, formal analysis. **Yuefeng Yin**: writing – review and editing, validation, software, formal analysis. **Nikhil V. Medhekar**: conceptualization, funding acquisition, writing – review and editing, project administration, supervision, resources.

## Conflicts of Interest

The authors declare no conflicts of interest.

## Supporting information




**Supporting File**: advs76833‐sup‐0001‐SuppMat.docx.

## Data Availability

The data that support the findings of this study are available on request from the corresponding author. The data are not publicly available due to privacy or ethical restrictions.
