# Supplementary material for: Flat Bands Induced by Non‐Collinear Antiferromagnetism in CoBi2Te4
Source: Adv Sci (Weinh). 2026 Jul 31;13(59):e76833. doi: 10.1002/advs.76833 (PMC13426398; doi:10.1002/advs.76833)
Supplement: Supplementary file 1 [file ADVS-13-e76833-s001.docx]

Supplementary Information

**Flat Band Induced by Non-collinear Antiferromagnetism in CoBi_2_Te_4_**

*Ziyuan Zhao^1,2*^, Yuefeng Yin^1,2^, Jinxing Gu^3^, Mark T. Edmonds^2,4^, Nikhil V. Medhekar^1,2,4*^*

^1^Department of Materials Science and Engineering, Monash University, Clayton, Victoria 3800, Australia

^2^ARC Centre of Excellence in Future Low-Energy Electronics Technologies FLEET, Monash University, Clayton, Victoria 3800, Australia

^3^Department of Chemical and Biological Engineering, Monash University, Clayton, Victoria 3800, Australia

^4^School of Physics and Astronomy, Monash University, Clayton, Victoria 3800, Australia

*Corresponding author: [nikhil.medhekar@monash.edu](mailto:nikhil.medhekar@monash.edu), [ziyuan.zhao@monash.edu](mailto:ziyuan.zhao@monash.edu)

**Index	Page**

1. Computational details………………………………………………………………………………2

2. Supplementary figures………………………………………………………………………………5

3. Supplementary tables………………………………………………………………………………13

4. References…………………………………………………………………………………………15

**Computational Details**

We carried out structural optimization as well as electronic and magnetic properties calculations using density functional theory (DFT)^1^ within the generalized gradient approximation (GGA)^2^ as implemented in the VASP code^3,4^. To account for van der Waals interactions, the DFT-D3 approach^5,6^ was applied. A vacuum spacing of approximately 15 Å was introduced to eliminate interactions between adjacent layers. The Hamiltonian contains scalar relativistic corrections and spin-orbit coupling (SOC) was explicitly included^7^. A plane-wave basis set with a cut-off energy of 500 eV was utilized for all calculations. The Monkhorst-Pack k mesh of 9 × 9 × 1 was used to yield a good convergence. All structural optimizations were performed using a conjugate-gradient algorithm, with a force convergence threshold of 0.01 eV/Å. To account for on-site electron-electron interactions in the Co 3*d*-states, a Hubbard *U* value of 4 eV was employed^8^. By evaluating the ground magnetic state of CoBi_2_Te_4_ 2SL under *U* values of 2, 4, and 6 eV, we verified the stability and robustness of the non-collinear antiferromagnetic (*nc*AFM) state as the ground magnetic state, as illustrated in Figure SI.

**Figure SI.** Energy difference per formula unit between different magnetic states of CoBi_2_Te_4_ 2SL as a function of different *U* values.

To search the ground magnetic state, we built FM and interlayer AFM configurations in unit cells, intralayer *c*AFM and *ncp*AFM configuration of 2 × 2 in-plane supercells containing four Co atoms per SL, and a triangular *nc*AFM configuration of $\sqrt{3}$ × $\sqrt{3}$ in-plane supercell containing three Co atoms per SL. The Monkhorst–Pack k-mesh of 5 × 5 × 1 and 4 × 4 × 1 were selected for the calculations of $\sqrt{3}$ × $\sqrt{3}$ and 2 × 2 supercells, respectively. And magnetocrystalline anisotropy energies (MAE) were calculated by using dense k-point meshes of 21 × 21 × 1 for unit cell. Since the MAE is sensitive to computational accuracy, we systematically tested its dependence on the *k*-mesh density and cutoff energy in AB-stacked CoBi_2_Te_4_ 2SL. As shown in Figure SII, the MAE stabilizes for *k*-meshes beyond 18 × 18 × 1, and the in-plane easy axis persists across cutoff energies ranging from 350 to 600 eV. Thus, the computational parameters used in this work, i.e., a *k*-mesh of 21× 21 × 1 and a cutoff energy of 500 eV) are well justified. To further verify the robustness of the MAE, we tested different exchange-correlation functionals. The calculated MAE values are -0.07 meV per Co (PW91^9^) and -0.05 meV per Co (PBEsol^10^), both confirming an in-plane easy axis in AB-stacked CoBi_2_Te_4_ 2SL. These consistent results across functionals strengthen the reliability of our findings.

**Figure SII** The MAE for AB-stacked CoBi_2_Te_4_ 2SL as a function of different *k* mesh (left) and cut-off energy (right).

We calculated the exchange coupling parameters between nearest neighbour Co atoms based on the Heisenberg model. The spin Hamiltonian is defined as

$\hat{H}$ = *E*_0_ -$\sum_{<ij>}^{N} J\vec{S}$*_i_*$\vec{S}$*_j_*

where *E*_0_ is the energy without magnetic coupling, the summation <*ij*> runs over all nearest-neighbour Co sites, *J* represents the exchange interaction between nearest-neighbour Co sites. The exchange parameter *J* is derived from the mapping analysis of FM, *c*AFM and *ncl*AFM. The Monte Carlo simulations were performed using a 16 × 16 supercell containing 768 spin sites. During the simulations, each spin rotates randomly in all directions. The critical temperature was finally obtained from the peak in the magnetic susceptibility.

Ab-initio-based tight-binding calculations were performed using the VASP package with the WANNIER90 interface^11,12^. The comparison of the DFT bands with tight-binding bands indicates that tight-binding model greatly replicates the electronic state (Figure SIII). The edge electronic band structure was calculated within the semi-infinite medium Green’s function approach^13,14^.

**Figure SIII** The comparison of the DFT bands with tight-binding bands obtained by the maximally localized Wannier functions considering the *d* orbitals of Co atoms and *p* orbitals of all Bi and Te atoms for CoBi_2_Te_4_ 2SLs.

**Supplementary Figures**

**Figure S1** Snapshot after equilibration (a) and free energy as a function of MD time (b) at the temperature of 300 K for CoBi_2_Te_4_ 1SL in the *nc*AFM configuration.

**Figure S2** Band structures with orbital characterization of CoBi_2_Te_4_ 1SL at the HSE level of theory excluding (a) and including SOC (b).

**Figure S3** The side views of AA and AB stacking configurations for CoBi_2_Te_4_ 2SL. The interlayer distances of AA and AB stacked configuration are 3.73 Å and 2.70 Å, respectively.

**Figure S4** The projected density of states (PDOS) for the *d*-orbitals of Co atom in CoBi_2_Te_4_ 2SL. The integrated PDOS of spin-up state on each *d*-orbital is ~1 eV, indicating each *d*-orbital is occupied by one spin-up electron. Another two electrons fractionally occupy the spin-down states, with priority in t_2g_ orbitals with lower energy.

**Figure S5** Four magnetic configurations of CoBi_2_Te_4_ 2SL considered here: ferromagnetic (FM) (a), intralayer collinear antiferromagnetic (intra_*c*AFM) (b), interlayer collinear antiferromagnetic (inter_*c*AFM) (c), and non-coplanar antiferromagnetic (*ncp*AFM) (d) configurations.

**Figure S6** Magnetic capacity of CoBi_2_Te_4_ 2SL obtained from Monte Carlo simulation. The dashed line represents the N$\acute{e}$el temperature.

**Figure S7** Band structures of CoBi_2_Te_4_ 1SL and AB-stacked 2SL under different *U* values of 2 eV and 6 eV.

**Figure S8** The Wannier Charge Center with close momentum plane for CoBi_2_Te_4_ 2SL.

**Figure S9** The top view of the relaxed (210) nanoribbon using DFT as implemented in the VASP code. The relaxed structure shows little atomic displacements no bond breakage at the edge.

**Figure S10** The edge band structures of left and right sides for the *nc*AFM terminations of the (210) nanoribbon under different *U* values of 2 eV and 6 eV.

**Figure S11** Band structures with Co-*d*, Bi-*p* and Te-*p* orbital characterization of AA-stacked CoBi_2_Te_4_ 2SL at the PBE level of theory excluding (a) and including SOC (b), respectively.

**Figure S12** (a) The top views of the atomic structure and the arrangement of Co atoms arrangement for the (210) nanoribbon of AA-stacked CoBi_2_Te_4_ 2SL. Orange and yellow colors represent Co atoms from the top and bottom SL, respectively; the arrows represent the magnetization directions of Co atoms. Note, since bottom later is directly beneath the top layer, the yellow atoms are not visible in the top view. (b) The edge band structure of (210) nanoribbon terminated by *nc*AFM coupling preserves a bulk band gap of 0.18 eV.

**Figure S13** (a) The top views of the atomic structure and the arrangement of Co atoms arrangement of the (210) nanoribbon with collinear magnetic terminations. The edge band structures of left and right sides for the collinear magnetic terminations of the (210) nanoribbon are shown in (b) and (c), respectively. Orange and yellow colors denote Co atoms from the top and bottom SL, respectively; the arrows represent the magnetization directions of Co atoms.

**Figure S14** Band structure with Co-*d*, Bi-*p* and Te-*p* orbital characterization of CoBi_2_Te_4_-Bi_2_Te_3_ heterostructure at the PBE level of theory excluding SOC.

**Figure S15** (a) Band structure of FeBi_2_Te_4_ 2SL with *nc*AFM state by considering SOC, showing band inversion between Bi-*p* and Te-*p* orbitals. (b) Edge state along the (210) direction of FeBi_2_Te_4_ 2SL, presenting flat bands near the Fermi level.

**Supplementary Tables**

**Table S1.** Structural information for CoBi_2_Te_4_ 2SL and CoBi_2_Te_4_-Bi_2_Te_3_ heterostructure.

|  | Lattice Parameter  *a* = *b* (Å) | Thickness  (Å) | Interlayer distance  (Å) |
| --- | --- | --- | --- |
| AB-stacked CoBi_2_Te_4_ 2SL | 4.26 | 23.93 | 2.70 |
| AA-stacked CoBi_2_Te_4_ 2SL | 4.25 | 25.04 | 3.73 |
| CoBi_2_Te_4_-Bi_2_Te_3_ | 4.30 | 20.80 | 2.65 |

**Table S2.** The relative energy differences of CoBi_2_Te_4_ 2SL and CoBi_2_Te_4_-Bi_2_Te_3_ heterostructure. Δ*E* is the energy differences of interlayer antiferromagnetic (inter_*c*AFM), intralayer antiferromagnetic (intra_*c*AFM), intralayer *nc*AFM and non-coplanar antiferromagnetic (*ncp*AFM) states relative to the ferromagnetic (FM) configuration.

|  | Δ*E*_inter__*_c_*_AFM_  (meV/Co atom) | Δ*E*_intra__*_c_*_AFM_  (meV/Co atom) | Δ*E_nc_*_AFM_  (meV/Co atom) | Δ*E_ncp_*_AFM_  (meV/Co atom) |
| --- | --- | --- | --- | --- |
| CoBi_2_Te_4_ 2SL | -5.81 | -14.08 | **-14.74** | -12.33 |
| CoBi_2_Te_4_-Bi_2_Te_3_ | - | -8.63 | **-9.78** | -7.55 |

**Table S3.** Specific edge energies (γ) for the (100), (010), and (210) edges of CoBi_2_Te_4_ 1SL. The specific edge energy is defined as *γ* = $\frac{E_{\mathrm{nanoribbon}}- nE_{\mathrm{layer}}}{2L}$, where *E*_nanoribbon_ is the total energy of nanoribbon, and *E*_layer_ is the energy per unit cell of CoBi_2_Te_4_ 1SL. *L* denotes the periodic length along the ribbon direction, and *n* is the number of unit cells contained in the nanoribbon supercell. The factor of 2 accounts for the presence of two edges in the nanoribbon.

| Nanoribbon | *E*_nanoribbon_ (eV) | *L* (Å) | *γ* (eV/ Å) |
| --- | --- | --- | --- |
| (100) | -414.95 | 7.38 | 0.75 |
| (010) | -415.35 | 7.38 | 0.72 |
| (210) | -409.25 | 12.79 | 0.65 |

**Table S4.** Bandwidths (*W*) and flatness ratios (*A*) of the flat bands in the *nc*AFM edge states along the (210) direction. The bandwidth is defined as *W* = *E*_max_ $-$ *E*_min_, and the flatness ratio is defined as *A* = $\Delta$ / *W*, where $\Delta$ is the energy gap between the dispersive edge bands above and below the flat band.

|  | *W* (meV) | *A* |
| --- | --- | --- |
| fb1 in *nc*AFM-left edge state | 23.91 | 2.17 |
| fb2 in *nc*AFM-left edge state | 38.53 | 1.35 |
| fb1 in *nc*AFM-right edge state | 17.63 | 2.95 |
| fb2 in *nc*AFM-right edge state | 37.46 | 1.39 |

**References**

(1)	Kohn, W.; Sham, L. J. Self-Consistent Equations Including Exchange and Correlation Effects. *Phys. Rev.* **1965**, *140* (4A), A1133–A1138.

(2)	Perdew, J. P.; Burke, K.; Ernzerhof, M. Generalized Gradient Approximation Made Simple. *Phys. Rev. Lett.* **1996**, *77* (18), 3865–3868.

(3)	Kresse, G.; Furthmüller, J. Efficient Iterative Schemes for Ab Initio Total-Energy Calculations Using a Plane-Wave Basis Set. *Phys. Rev. B* **1996**, *54* (16), 11169–11186.

(4)	Kresse, G.; Joubert, D. From Ultrasoft Pseudopotentials to the Projector Augmented-Wave Method. *Phys. Rev. B* **1999**, *59* (3), 1758–1775.

(5)	Grimme, S.; Antony, J.; Ehrlich, S.; Krieg, H. A Consistent and Accurate Ab Initio Parametrization of Density Functional Dispersion Correction (DFT-D) for the 94 Elements H-Pu. *J. Chem. Phys.* **2010**, *132* (15), 154104.

(6)	Grimme, S.; Ehrlich, S.; Goerigk, L. Effect of the Damping Function in Dispersion Corrected Density Functional Theory. *Journal of Computational Chemistry* **2011**, *32* (7), 1456–1465.

(7)	Koelling, D. D.; Harmon, B. N. A Technique for Relativistic Spin-Polarised Calculations. *J. Phys. C: Solid State Phys.* **1977**, *10* (16), 3107.

(8)	Anisimov, V. I.; Zaanen, J.; Andersen, O. K. Band Theory and Mott Insulators: Hubbard U Instead of Stoner I. *Phys. Rev. B* **1991**, *44* (3), 943–954.

(9)	Perdew, J. P.; Chevary, J. A.; Vosko, S. H.; Jackson, K. A.; Pederson, M. R.; Singh, D. J.; Fiolhais, C. Atoms, Molecules, Solids, and Surfaces: Applications of the Generalized Gradient Approximation for Exchange and Correlation. *Phys. Rev. B* **1992**, *46* (11), 6671–6687.

(10)	Perdew, J. P.; Ruzsinszky, A.; Csonka, G. I.; Vydrov, O. A.; Scuseria, G. E.; Constantin, L. A.; Zhou, X.; Burke, K. Restoring the Density-Gradient Expansion for Exchange in Solids and Surfaces. *Phys. Rev. Lett.* **2008**, *100* (13), 136406.

(11)	Silvestrelli, P. L.; Marzari, N.; Vanderbilt, D.; Parrinello, M. Maximally-Localized Wannier Functions for Disordered Systems: Application to Amorphous Silicon. *Solid State Communications* **1998**, *107* (1), 7–11.

(12)	Mostofi, A. A.; Yates, J. R.; Lee, Y.-S.; Souza, I.; Vanderbilt, D.; Marzari, N. Wannier90: A Tool for Obtaining Maximally-Localised Wannier Functions. *Computer Physics Communications* **2008**, *178* (9), 685–699.

(13)	Sancho, M. P. L.; Sancho, J. M. L.; Sancho, J. M. L.; Rubio, J. Highly Convergent Schemes for the Calculation of Bulk and Surface Green Functions. *J. Phys. F: Met. Phys.* **1985**, *15* (4), 851.

(14)	Henk, J.; Schattke, W. A Subroutine Package for Computing Green’s Functions of Relaxed Surfaces by the Renormalization Method. *Computer Physics Communications* **1993**, *77* (1), 69–83.
